# Nonlinear Microscopy of ECM Remodeling in Renal and Vascular Tissues: A Systematic Review Integrating Human AVF Imaging

**DOI:** 10.3390/medicina62020317

**Published:** 2026-02-03

**Authors:** Viltė Gabrielė Samsonė, Danielius Samsonas, Laurynas Rimševičius, Mykolas Mačiulis, Elena Osteikaitė, Birutė Vaišnytė, Edvardas Žurauskas, Virginijus Barzda, Marius Miglinas

**Affiliations:** 1 Faculty of Medicine, Institute of Clinical Medicine, Vilnius University, 01513 Vilnius, Lithuania; 2 Laser Research Centre, Faculty of Physics, Vilnius University, 01513 Vilnius, Lithuania; 3 Nephrology Centre, Vilnius University Hospital Santaros Klinikos, 08406 Vilnius, Lithuania; 4 Centre of Cardiology and Angiology, Clinic of Cardiac and Vascular Diseases, Vilnius University Hospital Santaros Klinikos, 08406 Vilnius, Lithuania; 5 State Pathology Center, Vilnius University Hospital Santaros Klinikos, 08406 Vilnius, Lithuania; 6 Department of Chemical and Physical Sciences, University of Toronto Mississauga, Toronto, ON L5L 1C6, Canada; 7 Department of Physics, University of Toronto, Toronto, ON M5S 1A1, Canada

**Keywords:** nonlinear microscopy, extracellular matrix, fibrosis, collagen remodeling, arteriovenous fistula

## Abstract

*Background and Objectives:* Extracellular matrix (ECM) and collagen remodeling contribute to chronic kidney disease (CKD) progression and vascular access dysfunction. Conventional histological techniques rely on staining and provide limited sensitivity for detecting early or subtle ECM alterations. Nonlinear optical imaging modalities, including second-harmonic generation (SHG), third-harmonic generation (THG), and multiphoton fluorescence (MPF) microscopy, enable label-free, high-resolution visualization of fibrillar collagen and may offer additional structural information. This study aimed to evaluate the added value of nonlinear imaging beyond conventional histology for assessing ECM remodeling in renal and vascular tissues. *Materials and Methods:* A systematic literature review was conducted in accordance with the PRISMA 2020 guidelines. PubMed and Web of Science were searched for studies published between 1 January 2015, and 4 April 2025, investigating ECM or collagen remodeling in renal or vascular tissues using SHG, THG, or MPF microscopy. After screening 115 records, 10 studies were included in the qualitative synthesis. In addition, representative SHG, THG, and MPF images of excised human arteriovenous fistula (AVF) tissue were acquired as illustrative feasibility examples to demonstrate the application of these imaging modalities. The use of human tissue was approved by the Vilnius Regional Biomedical Research Ethics Committee (approval No. 2022/6-1443-917). *Results:* The included studies demonstrated that nonlinear microscopy enables label-free assessment of collagen density, organization, and fiber orientation. SHG imaging differentiated healthy from diseased tissues and has been reported to support fibrosis assessment and staging in preclinical and selected clinical studies and revealed microstructural remodeling patterns not readily detected by conventional histology. The illustrative AVF images demonstrated collagen disorganization consistent with patterns reported in the reviewed literature and are presented solely to demonstrate imaging feasibility, without implying disease phenotype or clinical outcome associations. *Conclusions:* Nonlinear optical microscopy provides complementary structural information on ECM organization that is not accessible with standard histological techniques. Further validation and methodological standardization are required to support its broader application in clinical nephrology and vascular medicine.

## 1. Introduction

### 1.1. Extracellular Matrix Remodeling in Kidney Disease

Extracellular matrix (ECM) remodeling and collagen fiber organization contribute significantly to the pathophysiology of chronic kidney disease (CKD) and vascular access dysfunction [1]. In CKD, progressive ECM accumulation leads to structural disorganization of renal parenchyma, resulting in irreversible nephron loss [2,3,4]. Similarly, vascular access complications, particularly the maturation failure of arteriovenous fistulas (AVFs), are closely linked to fibrosis mediated impairment of vascular remodeling [4,5].

### 1.2. Nonlinear Optical Microscopy for ECM Assessment

To date, most routine assessments of ECM alterations rely on traditional histological techniques such as Masson’s trichrome or immunohistochemistry. However, these methods are inherently limited by subjectivity, dependence on staining protocols, and insufficient resolution to capture fine structural or orientation dependent features of collagen architecture. These shortcomings have accelerated interest in nonlinear optical microscopy, which offers a stain-free, high-resolution alternative for tissue evaluation [6,7]. SHG has emerged as a valuable imaging technique across biological systems, including kidney, liver, vascular, and neural tissues [8].

### 1.3. SHG-Based Assessment of Vascular ECM Remodeling

Beyond the kidney, SHG is used to evaluate AVF and vascular wall remodeling. Collagen disorganization detected by SHG correlates with AVF maturation outcomes and atherosclerotic risk. In arterial models, fiber orientation and mechanical integrity were associated with ECM changes visible by SHG. Similarly, studies in the brain, liver, and heart have employed SHG to track collagen alterations associated with ischemia, regeneration, and scarring processes [9,10]. SHG imaging has demonstrated progressive collagen fiber alignment during myocardial scar maturation, corresponding to changes in tissue mechanical properties [11,12]. SHG microscopy has shown that alterations in collagen organization, alignment, and polarity during cardiac scar formation reflect tissue anisotropy and healing integrity not captured by conventional histology [12,13]. SHG imaging has been used to examine structural remodeling of the vascular wall, revealing age- and diet-related shifts in aortic collagen fiber orientation as well as layer-specific differences in arterial stiffness linked to ECM microstructure [14,15]. Together, these findings highlight the value of SHG microscopy for label-free, real-time assessment of vascular ECM organization.

### 1.4. Evidence from Other Organ Systems: Contextual and Methodological Insights

Advances in nonlinear optical microscopy have further expanded the capabilities of SHG imaging for ECM assessment. Technical developments, such as the use of non-diffracting beam excitation, have improved imaging depth and collagen detection sensitivity, particularly in fibrotic tissues [16]. In hepatic disease models, combined SHG and multiphoton fluorescence imaging has enabled quantitative assessments of fibrosis progression and revealed age-dependent differences in collagen deposition patterns not detectable by conventional histology [17]. In oncology, SHG-based imaging has demonstrated that tumor-associated collagen organization, including fiber density and alignment, correlates with tumor grade, aggressiveness, and treatment response across multiple cancer types [18,19]. Multiphoton approaches have further revealed spatial heterogeneity of collagen architecture within tumor pseudocapsules and stromal compartments, with increased fibrosis linked to adverse clinical outcomes [20,21]. While not constituting core evidence for the present review, these studies provide important methodological context for the translational potential of nonlinear imaging in renal and vascular ECM remodeling.

### 1.5. Scope and Objectives of This Review

This systematic review synthesizes studies published between 2015 and 2025 on the application of SHG, THG, and MPF microscopy for assessing ECM remodeling, with a primary focus on renal and vascular tissues. The review evaluates the value of adding nonlinear imaging to conventional histology and identifies collagen-derived biomarkers relevant to fibrosis and vascular remodeling.

Selected examples from other organ systems are included solely for methodological context, while the qualitative synthesis and conclusions are explicitly anchored in renal and vascular ECM remodeling. Representative nonlinear microscopy images of human AVF tissue are presented to illustrate translational feasibility.

## 2. Materials and Methods

This systematic review was conducted in accordance with the PRISMA 2020 guidelines (see Supplementary Materials). A comprehensive and structured literature search was performed in PubMed/MEDLINE and the Web of Science Core Collection, covering studies published between 1 January 2015 and 4 April 2025. The search strategy combined terms related to nonlinear optical microscopy, including “nonlinear microscopy,” “second harmonic generation” (SHG), “third harmonic generation” (THG), and “multiphoton fluorescence” (MPF), with extracellular matrix-related terms such as “collagen” and “extracellular matrix” (ECM). These were combined with organ and disease-specific keywords including “kidney,” “renal,” “nephrology,” “artery,” “vein,” “blood vessel,” “vascular access,” “arteriovenous fistula,” and “dialysis.” Search terms were adapted to the indexing systems of each database.

After duplicate removal, two reviewers independently screened titles and abstracts for relevance. Full-text articles were retrieved when studies met the inclusion criteria or when eligibility could not be determined from the abstract alone. Discrepancies between reviewers were resolved by consensus. In addition, reference lists of included articles were manually screened to identify further relevant studies.

Eligible studies were original, peer-reviewed research articles published in English that applied SHG, THG, or MPF imaging to assess collagen organization or ECM remodeling in renal or vascular tissues. Both human and animal studies were eligible for inclusion. Reviews, editorials, conference abstracts, and studies not involving nonlinear microscopy were excluded.

From 115 records initially identified, 22 studies underwent full-text review. Of these, 10 methodologically comparable core studies specifically addressing renal and vascular ECM remodeling were included in the qualitative synthesis. Studies were considered methodologically comparable if they: (i) applied SHG-based imaging to renal or vascular tissues, (ii) directly assessed extracellular matrix or fibrillar collagen organization, and (iii) reported interpretable structural or quantitative outcomes relevant to fibrosis or vascular remodeling. Studies primarily focused on other organ systems or lacking ECM-specific outcomes were excluded from the core synthesis and referenced only for contextual and methodological comparison. The reviewed evidence encompassed murine, rat, sheep, and human tissues, with the qualitative synthesis explicitly focused on renal and vascular ECM remodeling. Studies involving other organ systems (e.g., cardiac, hepatic, cerebral, oncologic) were considered for contextual and methodological comparison only. Extracted data included biological sample type, nonlinear imaging modality, target tissue structures, and key findings related to fibrosis and extracellular matrix organization.

In addition to the literature review, original SHG, THG, and MPF images were acquired from surgically excised human AVF tissue samples as part of an observational imaging study. These images were included solely to illustrate the feasibility of multimodal nonlinear imaging in human vascular tissue and were not intended for quantitative analysis or clinical inference. Tissue acquisition and imaging were approved by the Vilnius Regional Biomedical Research Ethics Committee (approval No. 2022/6-1443-917). Written informed consent for the use of surgically excised tissue for research purposes was obtained from all patients prior to the surgical procedure.

The samples were fixed in 10% buffered formalin, stained with hematoxylin and eosin, sectioned, and imaged using a custom-built nonlinear laser-scanning microscope. A femtosecond oscillator (FLINT FL1, Light Conversion, Vilnius, Lithuania) delivering 100 fs pulses at a repetition rate of 76 MHz and a central wavelength of 1030 nm was used for excitation. The beam was raster-scanned using galvanometric mirrors (Saturn 5B, Pangolin Laser Systems, Orlando, FL, USA) and focused with a 20×/0.75 NA objective (Plan Apo Lambda, Nikon, Tokyo, Japan). Nonlinear signals were collected in transmission using a 0.45 NA singlet lens and detected with a photomultiplier tube (H10682-210, Hamamatsu Photonics, Hamamatsu, Japan) operating in photon-counting mode. Signal separation was achieved using optical filters placed in front of the detector: a 550 nm long-pass and 750 nm short-pass filter (Thorlabs Inc., Newton, NJ, USA) for MPF, a 10 nm band-pass filter centered at 515 nm (BP515-10, Edmund Optics, Barrington, NJ, USA) for SHG, and a 10 nm band-pass filter centered at 343 nm (FBH343-10, Thorlabs Inc., Newton, NJ, USA) for THG. Image visualization was performed using Fiji (ImageJ, version 1.54f (National Institutes of Health, Bethesda, MD, USA). No formal risk-of-bias or methodological quality assessment tool was applied due to the substantial heterogeneity in imaging platforms, biological models, and reported outcomes across studies.

## 3. Results

### 3.1. Study Selection

**Figure 1:**
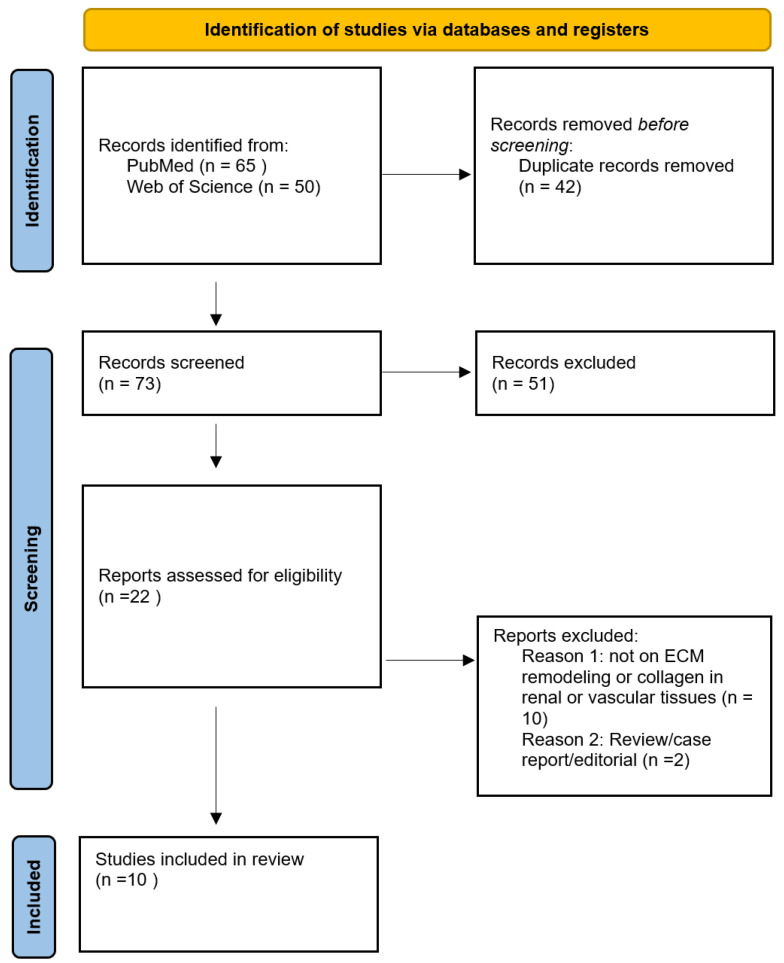
PRISMA flow diagram illustrating the literature search and selection process. Flowchart showing the number of studies identified, screened, included, and excluded at each stage of the systematic review process according to the PRISMA 2020 guidelines.

The database search retrieved 115 records—65 from PubMed/MEDLINE and 50 from Web of Science. After removal of 42 duplicates, 73 unique records were screened based on titles and abstracts. Of these, 22 articles underwent full-text review, and 10 studies met the inclusion criteria and were included in the final qualitative synthesis (Table 1). The study selection process is summarized in the PRISMA flow diagram in Figure 1.

### 3.2. Characteristics of Included and Contextual Studies

The characteristics and main findings of the included and contextual studies are summarized in Table 1. The reviewed studies encompassed murine, rat, sheep, rabbit, and human tissue samples. Renal applications primarily focused on chronic kidney disease models, kidney transplant assessment, and renal cell carcinoma pseudocapsule evaluation. Vascular applications included AVF development, arterial remodeling under mechanical stress, and diabetic vascular pathology.

All included core studies employed SHG as the primary modality for evaluating ECM remodeling or collagen architecture. Three studies additionally incorporated THG, and four studies used MPF imaging for complementary visualization.

### 3.3. Nonlinear Microscopy Findings in Renal and Vascular Tissues

In renal fibrosis models, SHG imaging enabled sensitive quantification of interstitial fibrosis, collagen fiber density, and orientation, which correlated with histological fibrosis scores and disease progression. In kidney transplant research, SHG analysis of donor biopsies quantified collagen content and organization and was associated with post-transplant outcomes. In renal cell carcinoma, SHG distinguished low- and high-grade tumors based on differences in collagen alignment and density.

In vascular studies, SHG microscopy revealed ECM disorganization associated with failed AVF maturation and vascular remodeling under mechanical stress. Collagen fiber realignment following interventions such as stenting or artery skeletonization was consistently detectable using SHG imaging.

### 3.4. Original Nonlinear Imaging of Human AVF Tissue

**Figure 2:**
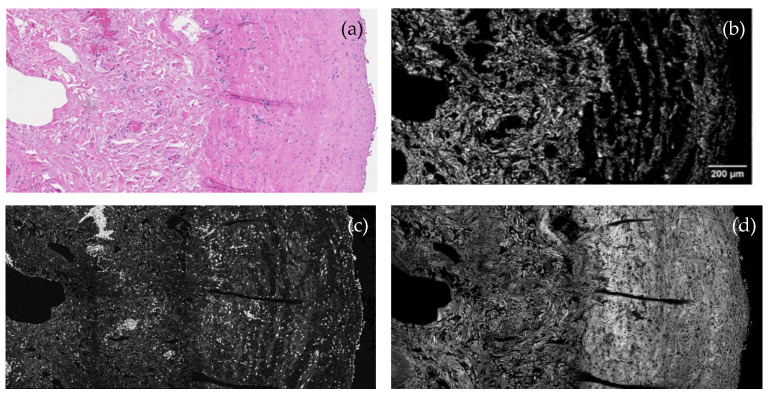
Representative nonlinear microscopy images of human arteriovenous fistula (V. cephalica) tissue. (**a**) Conventional light microscopy with hematoxylin and eosin (H&E) staining. (**b**) Second-harmonic generation (SHG) imaging highlighting fibrillar collagen organization predominantly within the media and adventitia (excitation: 1030 nm; detection: 515 ± 5 nm). (**c**) Third-harmonic generation (THG) imaging visualizing tissue interfaces and structural heterogeneities (detection: 343 ± 5 nm). (**d**) Multiphoton fluorescence (MPF) imaging revealing fluorescent tissue components (detection: 550–750 nm). Images are representative and illustrate collagen disorganization and altered vascular wall microstructure. Scale bar: 200 µm.

To complement the findings of the systematic review, original SHG, THG, and MPF images obtained from human AVF tissue are presented (Figure 2). These images demonstrate collagen fiber disorganization and altered microstructural organization. The observed patterns are consistent with remodeling features previously reported in SHG-based studies of AVF tissue.

### 3.5. Significance of Nonlinear Imaging in Human AVF Tissue

To complement the systematic literature review, representative multimodal nonlinear microscopy images of excised human arteriovenous fistula tissue were acquired. These images illustrate collagen fiber disorganization within the vascular wall, consistent with the remodeling patterns previously reported in SHG-based studies of AVF maturation failure.

No quantitative fiber orientation or alignment metrics were extracted from these images. Their inclusion serves to illustrate the translational feasibility of SHG, THG, and MPF imaging in human vascular tissue. The multimodal approach highlights complementary structural information beyond conventional histology, with SHG providing collagen-specific contrast, THG delineating tissue interfaces, and MPF visualizing fluorescent components.

These data should be interpreted as illustrative feasibility examples rather than definitive quantitative evidence of pathological remodeling. Although venous samples obtained prior to AVF creation can be considered representative of healthy veins, no formal control–disease group comparisons or quantitative analyses were performed within the scope of this systematic review. Accordingly, no claims of disease phenotype, AVF failure status, or clinical outcome association are made in this review.

## 4. Discussion

This systematic review highlights the diagnostic and research value of second-harmonic generation (SHG) and related nonlinear optical modalities for evaluating collagen architecture and extracellular matrix remodeling, with a primary focus on renal and vascular tissues.

In renal fibrosis models, SHG imaging consistently enabled sensitive and observer-independent detection of progressive fibrosis through quantitative assessment of collagen density, orientation, and microstructural organization [2,3,27]. Across experimental models of chronic kidney injury, SHG-based metrics outperformed conventional histological stains in capturing early and subtle fibrotic changes, supporting their utility for fibrosis staging and treatment response evaluation [2,3]. In human kidney tissue, multiphoton and SHG imaging differentiated healthy and diseased parenchyma and revealed pathological features such as glomerulosclerosis, tubular atrophy, and interstitial collagen remodeling without the need for exogenous staining [26]. Advances combining nonlinear microscopy with optical tissue clearing further enabled three-dimensional visualization of renal collagen networks and glomerular structures at millimeter-scale depths, highlighting the potential of these techniques for enhanced structural assessment of kidney pathology [28].

SHG has also demonstrated promise in donor kidney evaluation. Quantitative SHG-derived metrics of collagen amount and organization, such as collagen area ratio and reticulation indices, were associated with post-transplant outcomes, suggesting that nonlinear imaging may provide complementary information beyond conventional histological scoring by capturing fibrosis quality rather than quantity alone [22].

In vascular applications, SHG microscopy has provided important insights into ECM remodeling underlying arteriovenous fistula (AVF) maturation and vascular adaptation. Disordered collagen fiber orientation, reduced anisotropy, and increased medial fibrosis detected by SHG have been associated with failed AVF maturation in patients with chronic kidney disease [4,5]. In addition, SHG imaging has been used to evaluate therapeutic strategies aimed at preserving vascular collagen architecture, demonstrating improved AVF patency and structural integrity following targeted interventions [5].

Beyond AVF pathology, nonlinear imaging has characterized ECM remodeling following vascular interventions and under mechanical stress. SHG revealed collagen realignment along stent struts without changes in total collagen content after pulmonary artery stenting, underscoring the importance of microstructural organization rather than bulk collagen accumulation [23]. Similarly, stabilization of collagen architecture following artery skeletonization was associated with preserved vessel elasticity [29]. In models of atherosclerosis and diabetes, SHG captured dynamic remodeling processes, including circumferential collagen reorientation, adventitial bracing fiber formation, and layer-specific mechanical heterogeneity linked to disease progression and hemodynamic stress [24,25,30]. Age-related alterations in arterial collagen alignment and stiffness have also been demonstrated in human vascular tissue using SHG imaging [31].

Beyond renal and vascular tissues, selected studies provide important methodological context. In hepatic and cerebral models, SHG imaging has been applied to track dynamic collagen remodeling during regeneration and ischemic injury, illustrating the ability of nonlinear microscopy to resolve temporally evolving ECM alterations in vivo [32,33,34]. In oncology, SHG-based approaches have demonstrated that tumor-associated collagen organization, including fiber density, alignment, and spatial heterogeneity, correlates with tumor grade, aggressiveness, and progression across multiple cancer types, including renal cell carcinoma and colorectal cancer [18,19,20,21,35]. These findings further support the sensitivity of SHG-derived microstructural biomarkers for characterizing pathological ECM remodeling.

Collectively, the reviewed studies demonstrate that SHG and related nonlinear imaging techniques provide label-free, high-resolution insights into ECM remodeling across renal and vascular tissues. By quantifying collagen microarchitecture rather than relying solely on bulk collagen content, these modalities reveal structural features not readily accessible by conventional histology. Their integration with advanced imaging strategies and computational analysis could position nonlinear microscopy as a powerful tool for translational research and future clinical investigation in nephrology and vascular medicine.

Limitations: This review has several limitations. Despite comprehensive searches of PubMed and Web of Science, relatively few studies applied SHG, THG, or MPF specifically to renal or vascular ECM remodeling. To contextualize the broader potential of nonlinear microscopy, selected examples from oncology, hepatology, and neuroscience were included, introducing some heterogeneity. No formal risk-of-bias or methodological quality assessment tool was applied in this review. Due to substantial heterogeneity in imaging platforms, biological models, outcome measures, and reporting standards across studies, established tools such as QUADAS-2 or SYRCLE were not considered applicable. This limitation reduces the strength of comparative inference and highlights the need for standardized reporting and quality assessment frameworks in future nonlinear imaging studies. Many studies were preclinical, and human investigations often involved small cohorts. Reporting bias may also be present, as technically successful or positive studies are more likely to be published.

Future Directions: Future research should prioritize clinical validation of nonlinear imaging—particularly SHG—in larger, prospective, multicenter cohorts focusing on renal and vascular disease. Correlating imaging-derived biomarkers such as collagen alignment, polarity, and density with histopathology and clinical outcomes will be essential. Standardization of imaging protocols, calibration strategies, and analysis pipelines is required to enhance reproducibility and comparability. Advances that increase imaging depth, speed, and real-time feedback, together with integration of artificial intelligence and machine learning approaches, may enable intraoperative or bedside applications while improving pattern recognition, automating fibrosis quantification, reducing interobserver variability, and supporting the use of nonlinear microscopy as a dynamic biomarker for monitoring therapy response and ECM remodeling kinetics.

The illustrative AVF images presented in this study complement the existing SHG-based vascular access literature by demonstrating the feasibility of multimodal nonlinear imaging in human tissue. While prior studies primarily focused on SHG-derived quantitative metrics of collagen alignment and anisotropy, the present images emphasize the complementary structural context provided by THG and MPF. Although limited by the absence of quantitative analysis and clinical correlation, these images support the translational potential of nonlinear microscopy as an adjunct to conventional vascular pathology assessment.

## 5. Conclusions

This systematic review highlights the growing utility of nonlinear optical imaging—particularly SHG microscopy—as a sensitive, label-free approach for assessing ECM remodeling in renal and vascular tissues. Across preclinical and selected clinical studies, SHG consistently enabled quantitative evaluation of collagen fiber alignment, density, and polarity—structural features not accessible with conventional histological techniques but are highly relevant to fibrosis progression, vascular remodeling, and tissue integrity.

In nephrology, SHG demonstrated value for fibrosis staging, differentiation between healthy and diseased tissue, and assessment of collagen organization in transplant-related settings. In vascular research, SHG revealed collagen disorganization associated with AVF maturation failure and vascular remodeling processes. Together, these findings support the relevance of SHG-derived collagen metrics for studying ECM remodeling in renal and vascular disease.

By synthesizing current evidence and presenting illustrative SHG, THG, and MPF images of human AVF tissue, this review demonstrates the technical feasibility and translational relevance of nonlinear imaging in a clinically relevant vascular tissue context. While THG and MPF provided complementary structural information, SHG emerged as the most robust and consistently validated modality across renal and vascular studies.

At the same time, the available evidence remains limited by the relatively small number of human studies and the predominance of preclinical models. The findings summarized here should therefore be interpreted as demonstrating methodological potential and feasibility rather than immediate clinical applicability.

Despite remaining challenges, including methodological heterogeneity and lack of standardized protocols, the accumulated evidence positions nonlinear microscopy as a valuable adjunct to conventional histology and a promising research tool with potential future diagnostic applications. Clinical validation of these approaches is actively ongoing, including dedicated studies currently being conducted by our group; however, detailed outcome-based analyses fall beyond the scope of this systematic review and will be reported separately. Future efforts should focus on multicenter validation of imaging-derived biomarkers, standardization of acquisition and analysis workflows, and correlation with clinical outcomes to support broader clinical implementation in nephrology and vascular medicine.

## Figures and Tables

**Table 1:** Summary of studies applying SHG, THG, or MPF imaging for ECM assessment in renal and vascular tissues and related translational contexts. Overview of the study characteristics including sample types, imaging modalities used, biological targets investigated, and the key findings relevant to ECM remodeling and collagen organization. Studies were selected from the systematic literature review conducted for the period 2015–2025.

Study	Sample Type	Imaging Modality	Biological Target	Key Findings
Ranjit et al. [2]	Mouse kidney UUO	SHG + fluorescence lifetime imaging (FLIM)	Renal collagen	Improved quantification of renal fibrosis using label-free imaging.
Bhuiyan et al. [3]	Mouse kidney UUO	SHG	Renal collagen	SHG platform assessed antifibrotic therapy response and collagen morphology.
So et al. [22]	Human kidney	SHG + TPEF	Renal collagen	CART and CRI parameters correlated with donor kidney quality.
Allon et al. [4]	Human vein (AVF)	SHG	Vascular collagen	Disordered fiber orientation associated with AVF maturation failure.
Shiu et al. [5]	Rat vein (AVF)	SHG	Vascular collagen	Natural vascular scaffolding therapy improved AVF remodeling and collagen alignment.
Ghazanfari et al. [23]	Sheep pulmonary artery	SHG	Vascular collagen	Stents in arteries altered local collagen orientation; remodeling mapped via SHG.
Watson et al. [24]	Mouse aorta	SHG	Vascular collagen	Plaque formation drives collagen fiber reorganization in atherosclerosis.
Sugita S, Matsumoto T [25]	Rabbit thoracic aorta	SHG + Multiphoton microscopy (MPM)	Vascular collagen	Collagen fibers straighten under pressure, highlighting mechanical differences within the aortic wall.
Best et al. [18]	Human kidney—renal cell carcinoma (RCC) tissue	SHG	Renal collagen	Collagen density and alignment differed between low/high-grade RCC.
Sabo et al. [26]	Human kidney	SHG	Renal collagen	Label-free imaging accurately identifies kidney tissue quality and disease.

## Data Availability

Data supporting the findings of this study are available from the corresponding author upon reasonable request. Public sharing of the original human arteriovenous fistula imaging data is restricted due to ethical and privacy considerations.

## Supplementary Materials

The following supporting information can be downloaded at: https://www.mdpi.com/article/10.3390/medicina62020317/s1, The PRISMA 2020 reporting checklist.

## Abbreviations

The following abbreviations are used in this manuscript:

AVF	Arteriovenous fistula
CART	Collagen area ratio in tissue
CKD	Chronic kidney disease
CRI	Collagen reticulation index
ECM	Extracellular matrix
FLIM	Fluorescence lifetime imaging microscopy
H&E	Hematoxylin and eosin
MPF	Multiphoton fluorescence
PRISMA	Preferred Reporting Items for Systematic Reviews and Meta-Analyses
RCC	Renal cell carcinoma
SHG	Second-harmonic generation
THG	Third-harmonic generation
TPEF	Two-photon excitation fluorescence
