# Supplementary material for: Nonlinear Microscopy of ECM Remodeling in Renal and Vascular Tissues: A Systematic Review Integrating Human AVF Imaging
Source: Medicina (Kaunas). 2026 Feb 3;62(2):317. doi: 10.3390/medicina62020317 (PMC12943302; doi:10.3390/medicina62020317)
Supplement: Supplementary file 1 [file medicina-62-00317-s001.zip › medicina-4085720-supplementary.pdf]

The PRISMA 2020 reporting checklist

	Item Description	Location (or reason for not reporting)
**Title and Abstract**		
1. Title	Identify the report as a systematic review.	Title page (title includes "Systematic Review")
2. Abstract	Include all items from the *PRISMA 2020 for Abstracts* checklist.	Abstract, p. 1
**Introduction**		
3. Rationale	Describe the rationale for the review in the context of existing knowledge.	Introduction, p. 2-3
4. Objectives	Provide an explicit statement of the objective(s) or question(s) the review addresses.	End of Introduction, (final paragraph)
**Methods**		
5. Eligibility criteria	Specify the inclusion and exclusion criteria for the review and how studies were grouped for the syntheses.	Methods, p. 4
6. Information sources	Specify all databases, registers, websites, organisations, reference lists, and other sources searched or consulted to identify studies. Specify the date when each source was last searched or consulted<br>• Specify the date when each source (such as database, register, website, organisation) wa...	Methods, p. 4
7. Search	Present the full search strategies for all databases, registers, and websites, including any filters and limits used<br>• Provide the full line by line search strategy as run in each database with a sophisticated interface (such as Ovid), or the sequence of terms that were used to search si...	Methods, p. 4; Supplementary Table S1.
8. Selection Process	Specify the methods used to decide whether a study met the inclusion criteria of the review, including how many reviewers screened each record and each report retrieved, whether they worked independently, and, if applicable, details of automation tools used in the process.	Methods, p. 4
9. Data collection process	Specify the methods used to collect data from	Methods, p. 4

	reports, including how many reviewers collected data from each report, whether they worked independently, any processes for obtaining or confirming data from study investigators, and, if applicable, details of automation tools used in the process<br><br>• R...	
10. Data Items		
10a. Outcomes	List and define all outcomes for which data were sought. Specify whether all results that were compatible with each outcome domain in each study were sought (for example, for all measures, time points, analyses), and, if not, the methods used to decide which results to collect.<br><br>• List and de...	Methods, p. 4
10b. Other Variables	List and define all other variables for which data were sought (such as participant and intervention characteristics, funding sources). Describe any assumptions made about any missing or unclear information<br><br>• List and define all other variables for which data were sought. It may be sufficien...	Methods, p. 4
11. Risk of bias in individual studies	Specify the methods used to assess risk of bias in the included studies, including details of the tool(s) used, how many reviewers assessed each study and whether they worked independently, and, if applicable, details of automation tools used in the process.<br><br>• Specify the tool(s) (and version) u...	Not conducted – descriptive methodological review including heterogeneous preclinical and clinical imaging studies, for which no standardized risk-of-bias assessment tool is applicable.
12. Effect measures	Specify for each outcome the effect measure(s) (such as risk ratio, mean difference) used in the synthesis or presentation of results.<br><br>• Specify for each outcome or type of outcome (such as binary, continuous) the effect measure(s) (such as risk ratio, mean difference) used in the synthe...	Not applicable – narrative qualitative synthesis without effect size estimation.
13. Synthesis Methods		
13a. Deciding which studies were eligible for each	Describe the processes used to decide which studies were eligible for each synthesis (such as tabulating the study intervention characteristics and comparing	Methods, p. 4; Figure 1 (PRISMA flow diagram).

synthesis	against the planned groups for each synthesis described in item 5.	
13b. Data preparation methods	Describe any methods required to prepare the data for presentation or synthesis, such as handling of missing summary statistics or data conversions.<br>• Report any methods required to prepare the data collected from studies for presentation or synthesis, such as handling of missing summary…	Not applicable – descriptive synthesis only; no data transformations performed.
13c. Methods for tabulating or displaying results	Describe any methods used to tabulate or visually display results of individual studies and syntheses<br>• Report chosen tabular structure(s) used to display results of individual studies and syntheses, along with details of the data presented.<br>• Report chosen graphical methods used to v…	Table 1; Results section.
13d. Synthesis methods	Describe any methods used to synthesise results and provide a rationale for the choice(s). If meta-analysis was performed, describe the model(s), method(s) to identify the presence and extent of statistical heterogeneity, and software package(s) used.<br>• If statistical synthesis methods were used…	Not applicable – narrative descriptive synthesis only.
13e. Methods for exploring heterogeneity	Describe any methods used to explore possible causes of heterogeneity among study results (such as subgroup analysis, meta-regression).<br>• If methods were used to explore possible causes of statistical heterogeneity, specify the method used (such as subgroup analysis, meta-regression).<br>-…	Not applicable – no statistical synthesis performed
13f. Sensitivitiy analyses	Describe any sensitivity analyses conducted to assess robustness of the synthesised results.<br>• If sensitivity analyses were performed, provide details of each analysis (such as removal of studies at high risk of bias, use of an alternative meta-analysis model).<br>• If any sensitivity an…	Not applicable – descriptive synthesis; no sensitivity analysis conducted

14. Reporting bias assessment	Describe any methods used to assess risk of bias due to missing results in a synthesis (arising from reporting biases)<br>• Specify the methods (tool, graphical, statistical, or other) used to assess the risk of bias due to missing results in a synthesis (arising from reporting biases).<br>• …	Not applicable – no synthesis of effect sizes performed.
15. Certainty assessment	Describe any methods used to assess certainty (or confidence) in the body of evidence for an outcome<br>• Specify the tool or system (and version) used to assess certainty in the body of evidence.<br>• Report the factors considered (such as precision of the effect estimate, consistency of f…	Not applicable – no formal certainty assessment conducted; qualitative mapping of methods and findings
**Results**		
16. Study Selection		
16a. Results of the search and selection process	Describe the results of the search and selection process, from the number of records identified in the search to the number of studies included in the review, ideally using a flow diagram	Results, p. 5-7; Figure 1.
16b. Excluded studies	Cite studies that might appear to meet the inclusion criteria, but which were excluded, and explain why they were excluded.<br>• Cite studies that might appear to meet the inclusion criteria, but which were excluded, and explain why they were excluded.	Not reported – individual full-text exclusions were not listed due to the narrative nature of the qualitative synthesis.
17. Study characteristics	Cite each included study and present its characteristics.<br>• Cite each included study.<br>• Present the key characteristics of each study in a table or figure (considering a format that will facilitate comparison of characteristics across the studies).	Table 1; Results section.
18. Risk of bias in studies	Present assessments of risk of bias for each included study<br>• Present tables or figures indicating for each study the risk of bias in each	Not applicable – no formal risk-of-bias assessment conducted.

	domain/component/item assessed and overall study-level risk of bias.<br>• Present justification for each risk of bias judgment—for example, in t…	
19. Results of individual studies	For all outcomes, present for each study (*a*) summary statistics for each group (where appropriate) and (*b*) an effect estimate and its precision (such as confidence/credible interval), ideally using structured tables or plots<br>• For all outcomes, irrespective of whether statistical synthesis w…	Not applicable – descriptive synthesis of imaging findings only.
20. Results of Synthesis		
20a. Summary of studies	For each synthesis, briefly summarise the characteristics and risk of bias among contributing studies.<br>• Provide a brief summary of the characteristics and risk of bias among studies contributing to each synthesis (meta-analysis or other). The summary should focus only on study character…	Results section; Table 1
20b. Statistical results	Present results of all statistical syntheses conducted. If meta-analysis was done, present for each the summary estimate and its precision (such as confidence/credible interval) and measures of statistical heterogeneity. If comparing groups, describe the direction of the effect.<br>• Report results…	Not applicable – no statistical synthesis or group comparison; qualitative descriptive review only.
20c. Heterogeneity	Present results of all investigations of possible causes of heterogeneity among study results.<br>• If investigations of possible causes of heterogeneity were conducted:<br>○ present results regardless of the statistical significance, magnitude, or direction of effect modification. …	Not applicable – no investigations of heterogeneity performed due to narrative synthesis and diversity of included studies.
20d. Sensitivity analyses	Present results of all sensitivity analyses conducted to assess the robustness of the synthesised results<br>• If any sensitivity analyses were conducted:<br>○ report the results for each sensitivity	Not applicable – no sensitivity analyses conducted; qualitative synthesis only.

	○ analysis.<br>○ comment on how robust the main analysis was given the results of all…	
21. Risk of reporting biases in syntheses	Present assessments of risk of bias due to missing results (arising from reporting biases) for each synthesis assessed<br>• Present assessments of risk of bias due to missing results (arising from reporting biases) for each synthesis assessed.<br>• If a tool was used to assess risk of bias due …	Not applicable – no synthesis conducted; descriptive review without use of formal reporting bias assessment tools.
22. Certainty of evidence	Present assessments of certainty (or confidence) in the body of evidence for each outcome assessed<br>• Report the overall level of certainty in the body of evidence (such as high, moderate, low, or very low) for each important outcome.<br>• Provide an explanation of reasons for rating down (or…	Not applicable – no formal certainty assessment (e.g., GRADE) performed; qualitative review without outcome-specific grading.
**Discussion**		
23. Discussion		
23a. General interpretation of the results	Provide a general interpretation of the results in the context of other evidence<br>• Provide a general interpretation of the results in the context of other evidence.	Discussion.
23b. Limitations of included evidence	Discuss any limitations of the evidence included in the review<br>• Discuss any limitations of the evidence included in the review.	Discussion – Limitations subsection.
23c. Limitations of the review processes	Discuss any limitations of the review processes used<br>• Discuss any limitations of the review processes used and comment on the potential impact of each limitation.	Discussion – Limitations subsection.
23d. Implications	Discuss implications of the results for practice, policy, and future research<br>• Discuss implications of the results for practice and policy.	Discussion – Future Directions subsection.

	• Make explicit recommendations for future research.	
**Other Information**		
24. Registration and Protocol		
24a. Registration	Provide registration information for the review, including register name and registration number, or state that the review was not registered<br>• Provide registration information for the review, including register name and registration number, or state that the review was not registered.	The review was not registered.
24b. Protocol	Indicate where the review protocol can be accessed, or state that a protocol was not prepared<br>• Indicate where the review protocol can be accessed (such as by providing a citation, DOI, or link) or state that a protocol was not prepared.	No protocol was prepared.
24c. Amendments	Describe and explain any amendments to information provided at registration or in the protocol<br>• Report details of any amendments to information provided at registration or in the protocol, noting: (*a*) the amendment itself, (*b*) the reason for the amendment, and (*c*) the stage of the…	Not applicable – no protocol or registration.
25. Support	Describe sources of financial or non-financial support for the review, and the role of the funders or sponsors in the review<br>• Describe sources of financial or non-financial support for the review, specifying relevant grant ID numbers for each funder. If no specific financial or non-fina…	The authors received no external funding or assistance. Funding section.
26. Competing Interests	Declare any competing interests of review authors<br>• Disclose any of the authors' relationships or activities that readers could consider pertinent or to have influenced the review.<br>• If any authors had competing interests, report how they were managed for particular review processes.	Conflicts of Interest section.

27. Availability of data, code, and other materials	Report which of the following are publicly available and where they can be found: template data collection forms; data extracted from included studies; data used for all analyses; analytic code; any other materials used in the review<br><br>• Report which of the following are publicly available: templa…	Data Availability Statement.
